# Thoracoscopic lobectomy for a 4-day-old neonate with a large congenital pulmonary airway malformation: a case report

**DOI:** 10.1186/s13019-020-01174-3

**Published:** 2020-07-01

**Authors:** Jinxi Huang, Chaoming Zhou, Qiang Chen, Dianming Wu, Junjie Hong, Songming Hong

**Affiliations:** 1 Department of Cadiothoracic Surgery, Fujian Children’s Hospital, 18 daoshan Road, Fuzhou City, Fujian Province China; 2 Department of Pediatric Surgery, Fujian Provincial Maternity and Children’s Hospital, 18 daoshan Road, Fuzhou City, Fujian Province China; 3 Department of Cardiothoracic Surgery, Fujian Provincial Maternity and Children’s Hospital, 18 daoshan Road, Fuzhou City, Fujian Province China

**Keywords:** Thoracoscopic lobectomy, Neonate, Congenital pulmonary airway malformation, Congenital cystic adenomatoid malformation, Case report

## Abstract

**Abstract:**

Background: Congenital pulmonary airway malformation is a rare congenital lung lesion. Infants with large Congenital pulmonary airway malformation can present with a series of serious symptoms. Surgery is still the main treatment. Thoracoscopic lobectomy for neonates is rarely reported.

**Case presentation:**

The authors report a case of a congenital pulmonary airway malformation located in the left lower lung of a 4-day-old female infant. Prenatally, the cystic adenomatoid malformation volume ratio was 2.99 according to ultrasound scan. After birth, thoracoscopic lobectomy was performed to alleviate respiratory failure and mediastinal hernia. The patient’s clinical symptoms and the X-ray re-examination showed good postoperative recovery.

**Conclusions:**

The purpose of this study is to indicate that a safe and effective minimally invasive surgery for the giant congenital pulmonary airway malformation is feasible, even for infants only 4 days old.

## Background

Congenital pulmonary airway malformation (CPAM), formerly known as congenital cystic adenomatoid malformation (CCAM), is a rare developmental dysplastic lesion of the foetal tracheobronchial tree, and minimally invasive surgery for CPAM in children has become more common in recent year s[1]. Most children with CPAM can be diagnosed by prenatal ultrasound. After the neonatal period, the likelihood of the lesion becoming symptomatic has been reported to vary from 3 to 86 %[2]. A measurement of the CVR (cystic adenomatoid malformation volume ratio) is often performed. The CVR is calculated by taking the product of the three-dimensional measurements of the lesion (height×width×length) and 0.523 (the formula for the volume of an ellipse) and dividing this result by the head circumference. Crombleholme and colleagues first described the CVR and found that a measurement greater than 1.6 at the initial foetal ultrasound investigation predicted an increased risk of hydrops developing in a patient with a CPA M[3]. Open surgery and foetal surgery for CPAM have been reported in recent years, but rare cases of thoracoscopic lobectomy in neonates have been reported. In this case, the CVR measured in the foetal period increased to 2.99. This is the first report of thoracoscopic lobectomy performed on a neonate on the 4th day after birth.

## Case presentation

**Fig. 1:**
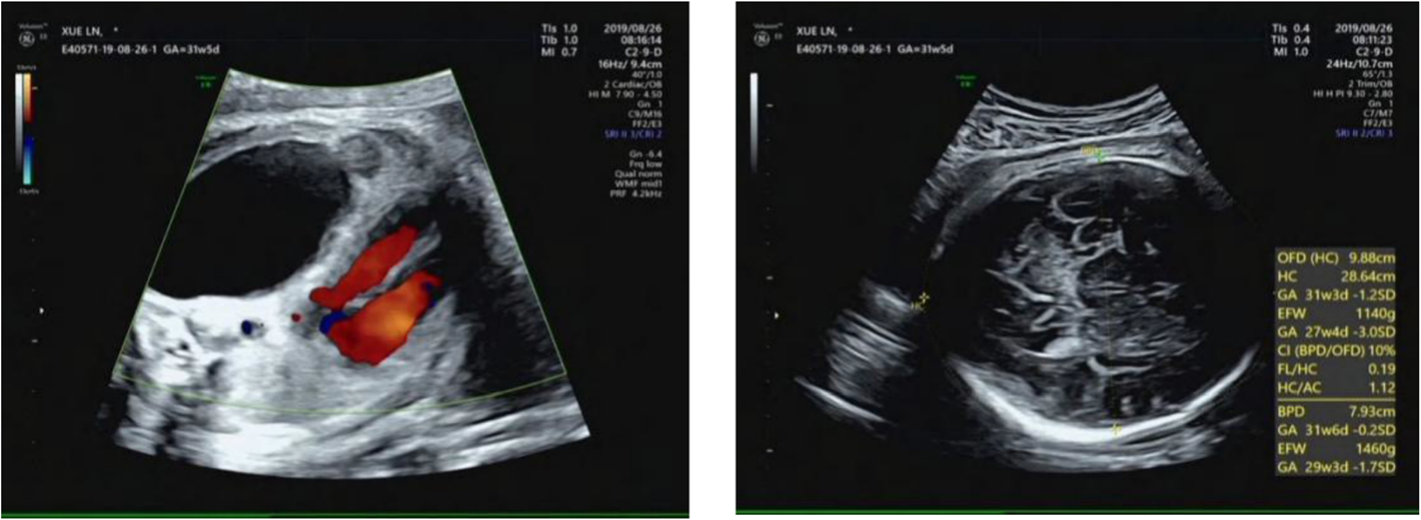
Antenatal ultrasound scan at 30 + 2 weeks gestation shows an echo-free cyst (5.4 cm × 3.3 cm × 5.6 cm) with a blood flow signal from the pulmonary circulation, a head circumference of 28.6 cm, and a CVR of 1.82

The child had healthy young parents and one older healthy sibling. At 30^+ 2^ weeks gestation, a routine antenatal ultrasound scan detected one echo-free cyst (5.4 cm × 3.3 cm × 5.6 cm) with a blood flow signal from the pulmonary circulation (Fig. 1). The CVR was 1.82.

**Fig. 2:**
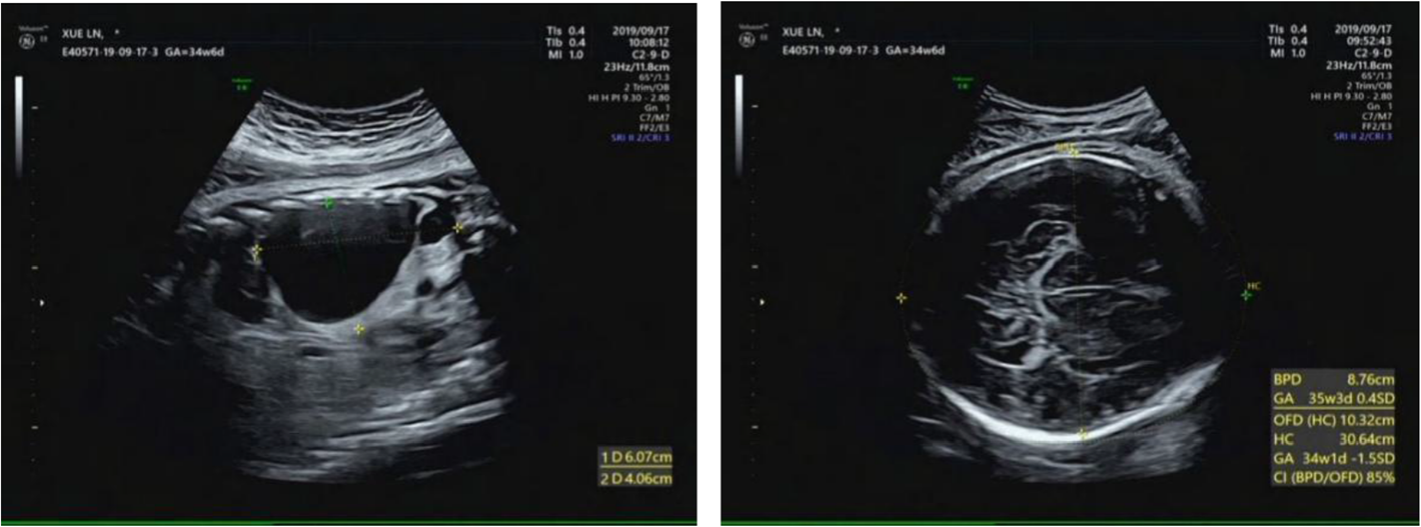
Antenatal ultrasound scan at 33 + 1 weeks gestation, and the ultrasound scan detected an echo-free cyst (6.1 cm × 4.1 cm × 5.9 cm) with a head circumference of 30.6 cm and a CVR of 2.52

**Fig. 3:**
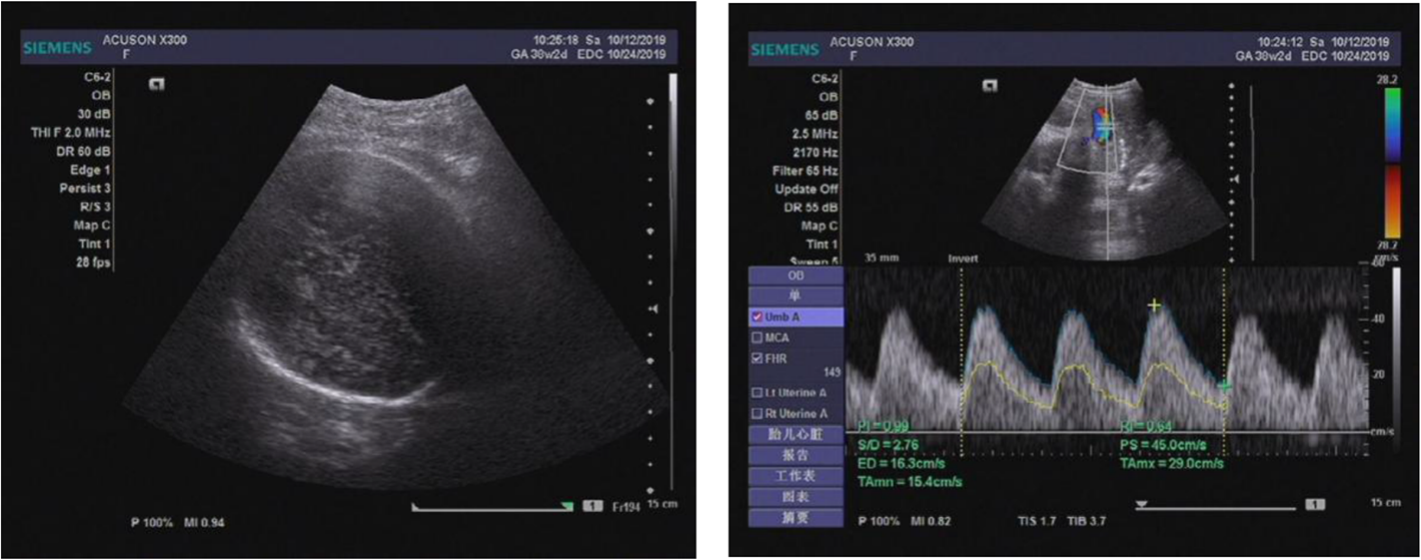
Antenatal ultrasound scan at 36 + 5 weeks gestation, and an ultrasound scan detected an echo-free cyst (5.0 cm × 5.4 cm × 6.8 cm) with a head circumference of 32.1 cm and a CVR of 2.99

The abnormality was judged as consistent with a macrocystic congenital cystic adenomatoid malformation of the lung situated in the left lower thoracic cavity, and this abnormality led to a significant shift of the mediastinal structures to the right side. Repeated ultrasound scans during pregnancy showed that the malformation grew (Figs. 2, 3).

After multidisciplinary consultation, early labour induction was recommended to the gravida, but she decided to continue her pregnancy. Finally, she had a vaginal delivery at 39 weeks gestation, and her birth weight was 2499 g.

**Fig. 4:**
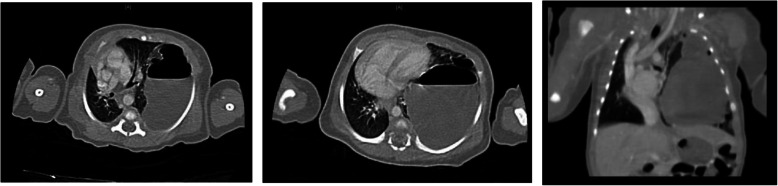
The CT scan showed that the cyst had caused severe compression of the heart, lungs, and mediastinum

After birth, the patient showed shortness of breath and progressive aggravation to respiratory failure. Oxygen given through a hood could not correct the hypoxemia of the infant. Four hours after birth, the infant underwent endotracheal intubation and ventilator-assisted breathing. The CT scan showed that the cyst had caused severe compression of the heart, lungs, and mediastinum (Fig. 4). The symptoms could not be controlled with ventilator-assisted breathing or other treatments, and dyspnoea became worse on the 4th day after birth. A left lower lung thoracoscopic lobectomy was suggested, and consent was obtained from the parents.

**Fig. 5:**
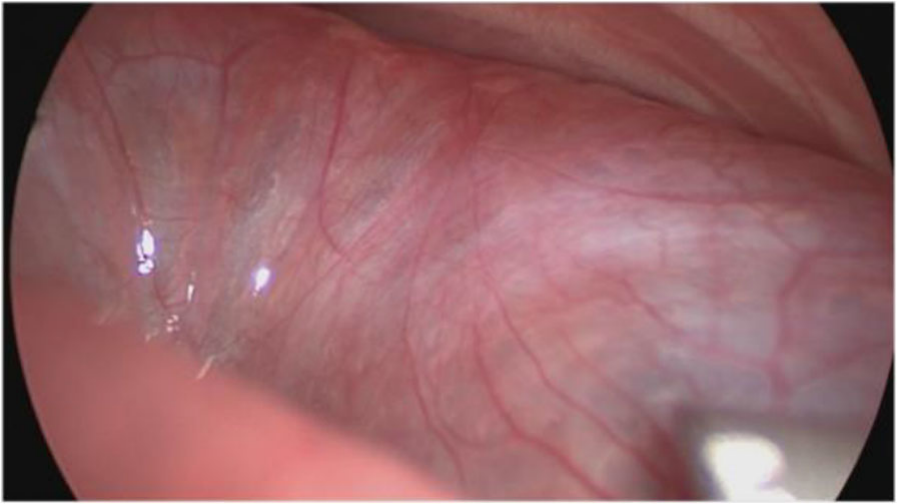
The left thoracic cavity is filled with a giant CCAM

**Fig. 6:**
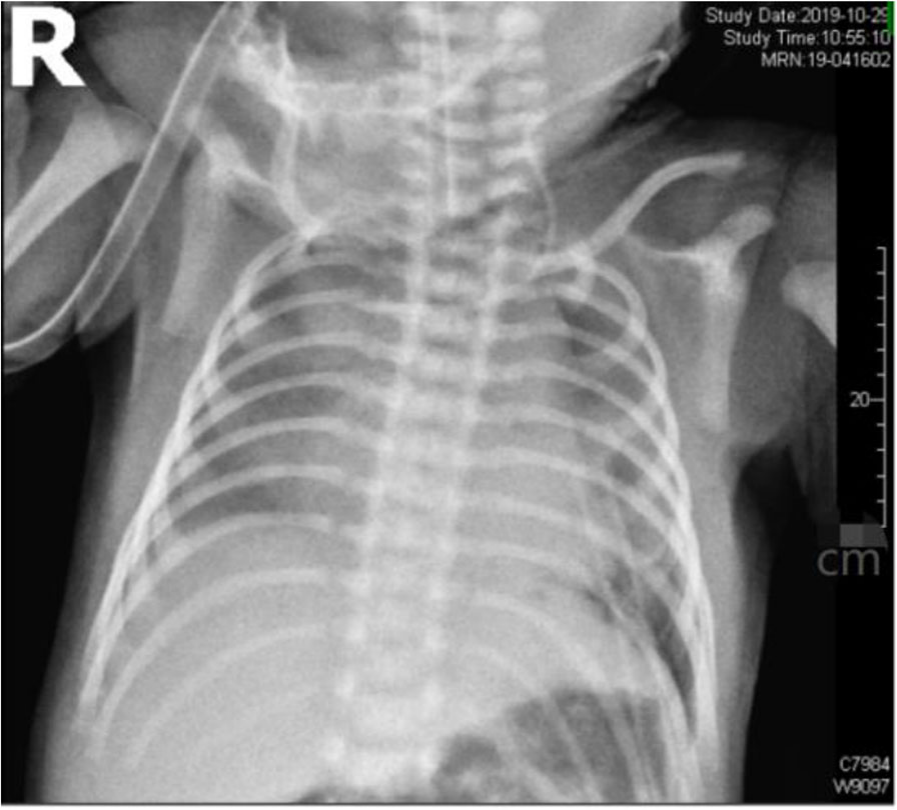
X-ray showing that the left lung recovered satisfactorily

No patients younger than this case were found in the existing literature for thoracoscopic lobectomy. The respiratory circulation of this child was difficult to maintain, so surgical treatment was selected. Thoracoscopy was performed at 4 days after birth. The patient was positioned on the right side with the left arm elevated. Two 5-mm trocars and one 3-mm trocar were used. One 5-mm trocar was positioned in the 8th intercostal space of the midaxillary line as the observation trocar, another 5-mm trocar was positioned in the 7th intercostal region of the posterior axillary line, and the 3-mm trocar was positioned in the 5th intercostal region of the anterior axillary line after artificial pneumothorax. The left thoracic cavity was filled with a giant CPAM (Fig. 5), and ultrasonic scalpel was used to open the cyst to make it smaller in order to expose the surgical field. The lower left lung was filled with several cystic structures. Hem-O-lock ligating clips were used to ligate the vessels. Clipping and division of the vessels and fissure followed by complete excision were performed, and the specimen was extracted through the 5-mm port site in the midaxillary line after spreading. The operation lasted 170 min. Histology confirmed the diagnosis of a CPAM. The postoperative course was uneventful. X-ray showed that the left lung recovered satisfactorily (Fig. 6). The child was extubated 6 days after surgery and discharged from the hospital 16 days after surgery. After surgery, the patient’s respiratory function and circulatory function recovered well. At 6 months post surgery, the child had no respiratory discomfort and performed normal activities, and no obvious abnormality was found upon reexamination of the chest radiograph.

## Discussion

Infants with CPAM can present in a wide range of severity, from being asymptomatic until later in life to having respiratory distress while in the neonatal perio d[4]. To date, several reports have stated that CVR > 1.6 can be used as a prognostic tool to predict the development of foetal complications or respiratory distress at birth [5–7] and that CVR > 1.0 could be considered a predictor of hydrops fetalis or early surgery at birt h[8].

Video-assisted thoracic surgery (VATS) has been shown to be a safe and effective technique for the diagnosis and treatment of paediatric pulmonary disease [9, 10]. Thoracoscopic lobectomy is still the main surgical treatment for CPAMs.

The child in this case had an incomplete fissure, and after cutting off the inferior pulmonary vein, we found it difficult to expose the inferior pulmonary artery, and the pulmonary fissure appeared to bleed easily. Therefore, we chose to cut it from the periphery of the pulmonary fissure with an ultrasonic scalpel, dissecting it to the place near the inferior pulmonary artery, and then ligate the lower pulmonary artery and the lower pulmonary bronchus with No. 0 silk suture. After ligation, the lower lung tissue was removed; thus, there was no bleeding or air leakage in the section. A review of the X-ray results demonstrated no residual cysts.

## Conclusions

With the development of thoracoscopic instruments and technologies, the age at which neonates can undergo thoracoscopic surgery is decreasing. In previous studies, foetal surgery, aspiration of the cyst and intrapartum foetal operation were recommended to intervene in large CPAMs. This case showed that thoracoscopic lobectomy for a large neonatal CPAM was feasible and effective in a 4-day-old neonate.

## Data Availability

Written informed consent was obtained from the patient for publication of this case report and any accompanying images. A copy of the written consent is available for review.

## Abbreviations

CPAM: Congenital pulmonary airway malformation
CVR: Cystic adenomatoid malformation volume ratio
CCAM: Congenital cystic adenomatoid malformation
VATS: Video-assisted thoracic surgery
CT: Computed tomography
